# Sanatorium Results

**Published:** 1920-01-31

**Authors:** 


### SANATORIUM RESULTS.

Careful account keeping, in one form or another, is a necessity in any undertaking, as even revolutionaries acknowledge. The profit side of the anti-tuberculosis campaign is an interesting study, much easier to make out in the case of some items than of others. In a recent publication* we get an orderly, orthodox stocktaking of the results of treatment of patients at the King Edward VII. Sanatorium, from its commencement. The work has been facilitated by a businesslike method of recording cases, and by the engagement of clerical help for the purpose of tracing after-histories of all patients treated since 1906. Only between three and four per cent. were lost sight of, which is very creditable. The inquiry follows lines adopted in a Galton Eugenics Laboratory examination of American and Scotch tuberculosis statistics by two actuaries (in the present instance an actuary collaborates with a physician). Otherwise there is but little reference to the literature of the subject, an omission whereby the memoir suffers. It would have ranked a good deal higher had a digest been made of the extensive existing European literature, and the requisite comparisons made, corroboratory or the reverse. The chief conclusions reached are, firstly, that, of course, mortality varies directly, so to say, with the amount of progress of disease on the patient's admission. This is well accepted. Then tuberculin treated patients did not do significantly better than others; and this finding is also in accord with the current trend of research and opinion. Again, it has long been taught that an initial hæmoptysis is in the nature of a blessing in disguise, frightening a patient into going to the doctor when in presence of a more insidious onset he might have fatally procrastinated. Corroborative statistical evidence is adduced that cases of such initial onset do better than others. Another piece of sanatorium lore was that it was a good thing to "get rid of your bacilli," *i.e.* to have your sputum found free from *B. tuberculosis* when it had contained this organism at the commencement of treatment; very favourable cases would eliminate them in the third month. This maxim also receives confirmation, and to direct inquiry to this detail was probably an improvement upon the comparison the Galton Eugenics inquiries had drawn between all blood-spitting cases and other consumptives. Similarly it is confirmed that laryngeal tubercle is a grave complication, while the many workers who have recorded that a family history of the disease is no guide to prognosis have another successor. The only serious fault of the production appertains to the high politics of it, a sphere where probably the authors wielded no great sway. Why was not material available, at a leading British sanatorium, for the testing of more up-to-date means of specific treatment? Tuberculin seems to have been introduced only in 1912, a date at which it was already sinking in the estimation of experts abroad as a therapeutic agent, after years of trial. 1912 would have been late to begin trying IK, which has practically been the immediate successor of tuberculin, and which is also now moribund. But in 1912 Dr. Lillingston was introducing from Scandinavia into Britain lung collapse therapy, an important and more promising measure which a German tuberculosis *Centralblatt* was publishing special numbers on in 1909. Other home sanatoria are now giving figures as to this treatment, yet we believe that the sanatorium this report hails from has not tried it even yet. In short, it looks like a case of "Wake up, Midhurst!" The report is issued as a brochure, and it is a pity that a paper jacket is not adopted for bigger books too. *La vie chère* is unfortunately with us, and there is no money to waste on stiff bindings, except for classics, library books, fine literature, and those very few volumes one is always re-reading. Scientific works now, here as they are already abroad, should be all "brosch." If they develop an unexpectedly attractive quality, it is easy to get them bound in boards.

* National Health Insurance Medical Research Committee, special Report Series No. 33. *Pulmonary Tuberculosis: Mortality after Sanatorium Treatment.* H.M. Stationery Office, 1919. Price 2s.

